# International Medical College

**Published:** 1876-06

**Authors:** 


INTERNATIONAL MEDICAL CONGRESS.—At the recent meeting of the Medical Association of Georgia, there were a number of delegates appointed to the above-named congress, to assemble in Philadelphia, September 4th. The occasion will be one of very great interest. It is hoped that each delegate will attend. Should any one find that he will not be present, he will confer a favor by so notifying Dr. J. G. Thomas, Savannah, in order that the privilege may be extended to some one in his stead.

The names of the delegates were announced in our last issue.

